# Alteration of proliferation and apoptotic markers in normal and premalignant tissue associated with prostate cancer

**DOI:** 10.1186/1471-2407-6-73

**Published:** 2006-03-17

**Authors:** Vijayalakshmi Ananthanarayanan, Ryan J Deaton, Ximing J Yang, Michael R Pins, Peter H Gann

**Affiliations:** 1 Department of Pathology, University of Illinois at Chicago, USA; 2 Department of Pathology, Feinberg School of Medicine, Northwestern University, Chicago, IL, USA; 3 Robert H. Lurie Comprehensive Cancer Center, Feinberg School of Medicine, Northwestern University, Chicago, IL, USA

## Abstract

**Background:**

Molecular markers identifying alterations in proliferation and apoptotic pathways could be particularly important in characterizing high-risk normal or pre-neoplastic tissue. We evaluated the following markers: Ki67, Minichromosome Maintenance Protein-2 (Mcm-2), activated caspase-3 (a-casp3) and Bcl-2 to determine if they showed differential expression across progressive degrees of intraepithelial neoplasia and cancer in the prostate. To identify field effects, we also evaluated whether high-risk expression patterns in normal tissue were more common in prostates containing cancer compared to those without cancer (supernormal), and in histologically normal glands adjacent to a cancer focus as opposed to equivalent glands that were more distant.

**Methods:**

The aforementioned markers were studied in 13 radical prostatectomy (RP) and 6 cystoprostatectomy (CP) specimens. Tissue compartments representing normal, low grade prostatic intraepithelial neoplasia (LGPIN), high grade prostatic intraepithelial neoplasia (HGPIN), as well as different grades of cancer were mapped on H&E slides and adjacent sections were analyzed using immunohistochemistry. Normal glands within 1 mm distance of a tumor focus and glands beyond 5 mm were considered "near" and "far", respectively. Randomly selected nuclei and 40 × fields were scored by a single observer; basal and luminal epithelial layers were scored separately.

**Results:**

Both Ki-67 and Mcm-2 showed an upward trend from normal tissue through HGPIN and cancer with a shift in proliferation from basal to luminal compartment. Activated caspase-3 showed a significant decrease in HGPIN and cancer compartments. Supernormal glands had significantly lower proliferation indices and higher a-casp3 expression compared to normal glands. "Near" normal glands had higher Mcm-2 indices compared to "far" glands; however, they also had higher a-casp3 expression. Bcl-2, which varied minimally in normal tissue, did not show any trend across compartments or evidence for field effects.

**Conclusion:**

These results demonstrate that proliferation and apoptosis are altered not only in preneoplastic lesions but also in apparently normal looking epithelium associated with cancer. Luminal cell expression of Mcm-2 appears to be particularly promising as a marker of high-risk normal epithelium. The role of apoptotic markers such as activated caspase-3 is more complex, and might depend on the proliferation status of the tissue in question.

## Background

Disturbances of proliferation and apoptosis are fundamental events in early carcinogenesis, and may be useful in characterizing tissue that is histologically normal but at high-risk for neoplastic growth. Field effect – referring to genetically altered but phenotypically normal-looking cells in the vicinity of a cancer focus [1-3] – has been studied fairly extensively in sites such as the oral cavity and lung.[4,5] Field effects have not been well characterized as yet in the prostate, although identification of these events could play an especially profound role in chemoprevention research and clinical practice. Due to the validity limitations of PSA testing and the lack of imaging tools which make blind biopsies necessary, benign prostatic tissue is sampled very commonly. In the U.S., approximately 1.5 million prostate biopsies are performed annually, and the great majority are negative for cancer.[6] All biopsies are likely to contain normal-appearing epithelium that could potentially harbor changes characteristic of high-risk changes at the genetic, protein, cytomorphological level. Therefore, biomarkers identifying high-risk non-cancer tissue could be very useful as intermediate endpoints in chemoprevention studies, and as tools for classifying patients with negative biopsies according to their need for close follow-up.

In this study, we analyzed the expression of the proliferation markers Ki67 and Mcm-2, the apoptotic marker a-casp3 and the anti-apoptotic marker Bcl-2. First, we evaluated whether these markers showed differential expression across progressive degrees of intraepithelial neoplasia and cancer. Because proliferation occurs predominantly in the basal layer, and apoptosis in the luminal layer of the normal prostatic epithelium, we determined whether early dysplastic progression was associated with a detectable shift towards proliferation and suppressed apoptosis in the luminal compartment. In order to address field effects in prostatic carcinogenesis, we first determined whether normal tissue from prostates that harbored a focus of cancer had different proliferative and apoptotic characteristics than normal tissue from prostates that did not contain cancer (designated as "supernormal"). Finally, we analyzed normal and HGPIN glands adjoining a focus of cancer to determine if cells within these epithelial structures showed high-risk expression patterns when compared to similar cells that are distant from a cancer focus.

## Methods

### Subjects and specimens

Thirteen RP specimens containing prostate cancer and six CP samples not containing prostate cancer were retrieved from Northwestern Memorial Hospital's pathology repository after appropriate Institutional Review Board approval. The radical prostatectomy specimens were procured from prostate cancer cases with Gleason scores ranging from five to nine. The most representative block(s) containing compartments of normal, LGPIN, HGPIN, and cancer were selected for further analysis. Although a diagnosis of LGPIN is not used in clinical reports, established criteria for identifying acinar structures with LGPIN can be used in research studies. LGPIN lesions were defined based on the following nuclear features: variation in nuclear size, nucleomegaly, normal or slightly increased chromatin content, and small or inconspicuous nucleoli.[7,8] HGPIN lesions had large nuclei with very prominent nucleoli. Both LGPIN and HGPIN lesions had either an intact or an attenuated basal cell layer. Cancerous areas were further subcategorized into three compartments: low-grade cancer (LGCA), intermediate grade cancer (IGCA) and high grade cancer (HGCA) based on Gleason grades 1–2, 3 and 4–5 respectively. Tissue representing all six compartments was mapped out on hematoxylin and eosin (H&E) stained slides using marker pens. In addition, normal areas were mapped for six cystoprostatectomy specimens obtained from patients undergoing treatment for bladder cancer. Prostates from cystoprostatectomy specimens were examined grossly for suspicious areas, which if present, were submitted for histopathologic examination. Otherwise, a minimum of four random sections were taken. All sections were studied in detail by a pathologist (MRP) to rule out the presence of cancer in the prostate.

### Immunohistochemistry

Five unstained sections were cut from each sample. The first section was stained by H&E and subsequent sections were immunostained for Ki67 (Dako, 1:200), Mcm-2 (Novocastra, 1:40), a-casp3 (Cell Signaling, 1:400), and Bcl-2 (Dako, 1:200) using a Dako autostainer. For all the antibodies, antigen retrieval was carried out in a steamer using Target Retrieval Solution (S1699, Dako, Carpinteria, CA). After treating with the appropriate antibody, sections were incubated with a ready-to-use anti-mouse secondary antibody from Dako (EnVision Plus^®^) and color reaction was developed using diaminobenzidine (DAB) as the chromagen. The slides were then counterstained with hematoxylin. Suitable positive and negative controls were run in tandem.

### Scoring and statistical analysis

#### Progressive compartments comparison

Areas corresponding to the marked regions on the H&E slides were mapped onto the immunostained slides. Ki67 and Mcm-2 immunostained slides were mounted with photo-etched cover slips (Bellco Glass Inc.). These cover slips have 520 alphanumeric squares (grids) etched on them, each measuring 0.6 × 0.6 mm. At the outset, the grid locations for supernormal, normal, LGPIN, HGPIN, LGCA, IGCA, and HGCA were recorded. For supernormal, normal, HGPIN, and cancer compartments, grids were presented in a random order in a Microsoft Access^® ^database for scoring. Strongly positive basal and luminal cells were evaluated separately for all compartments except cancer. A mean of 2,642 (range: 132–3,929) nuclei per slide was counted for these compartments. All LGPIN areas were evaluated. For a-casp3 and Bcl-2, only the luminal compartment was scored, as scoring basal cell cytoplasmic staining intensity is presumed to be too unreliable. Both intensity of immunostaining and percentage of immunopositive area were recorded at 40 × magnification. Intensity was recorded on an ordinal scale of 0–3, wherein 0 indicated absent staining, while 3 indicated intense staining. For each 40 × field, the percent of area positive for scores 0–3 was recorded. The sum of the product of percent positive area and intensity gave the final score for the 40 × field. For a-casp3 and Bcl-2, a mean of 15 (1–33) 40 × fields was scored. Table 1 gives information about the minimum number of nuclei/40 × fields evaluated for each marker across the RP and CP samples.

#### "Near" and "far" comparison

In order to compare normal and HGPIN glands near a focus of cancer versus those farther away, all tumor foci were initially mapped onto the H&E slide using colored marking pens. Different colors were used to mark out normal and HGPIN areas that were 1 mm, 5 mm and 10 mm away from tumor. Glands that were in close proximity and within 1 mm distance of the tumor were considered to be "near" and glands that were at least 5 mm distance away were considered to be "far". Glands at least 10 mm away from tumor were preferentially evaluated whenever available. These maps were subsequently traced out on the corresponding immunostained slides. For Ki67 and Mcm-2, a mean of 941 nuclei (maximum of 1264) per slide were randomly sampled from near and far, normal and HGPIN areas. For a-casp3 and Bcl-2, all "near" 40 × fields and at least ten "far" 40 × fields were scored. Glands were scored as described above.

#### Data analysis

Progressive compartment comparison, as well as "near" and "far" comparison was done by computing means with 95% confidence intervals and t statistics. All statistical analyses were performed using SAS^® ^version 9.1(Cary, NC). Pearson and Spearman correlation coefficients were used to examine correlations between Ki67 and Mcm-2.

## Results

### Biomarker expression across progressive tissue compartments

#### Ki67 and Mcm-2

**Figure 1:**
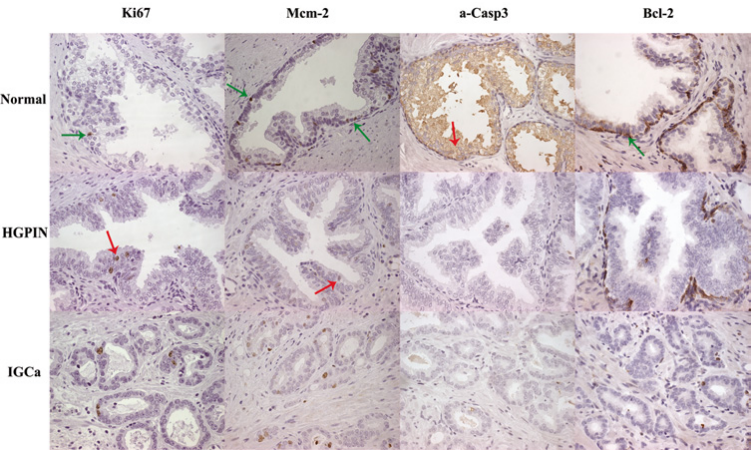
Figure 1 shows the expression of proliferation and apoptotic markers in the various compartments of the prostate. The immunomarkers are shown in columns from left to right (Ki67, Mcm-2, a-Casp3, and Bcl-2) and the compartments are shown in rows from top to bottom (Normal, HGPIN and IGCA). Normal compartment showed predominant basal cell (green arrows) positivity for Ki67 and Mcm-2. A shift towards the luminal compartment (red arrows) is noted for these two proliferation markers in HGPIN. Activated Caspase-3 on the other hand, showed a progressive decrease in the luminal cell staining from normal through PIN to cancer. Bcl-2 did not show any differential trend across compartments. Note that the strongly positive basal cells (for Bcl-2) serve as positive internal controls for the assay.

**Figure 2:**
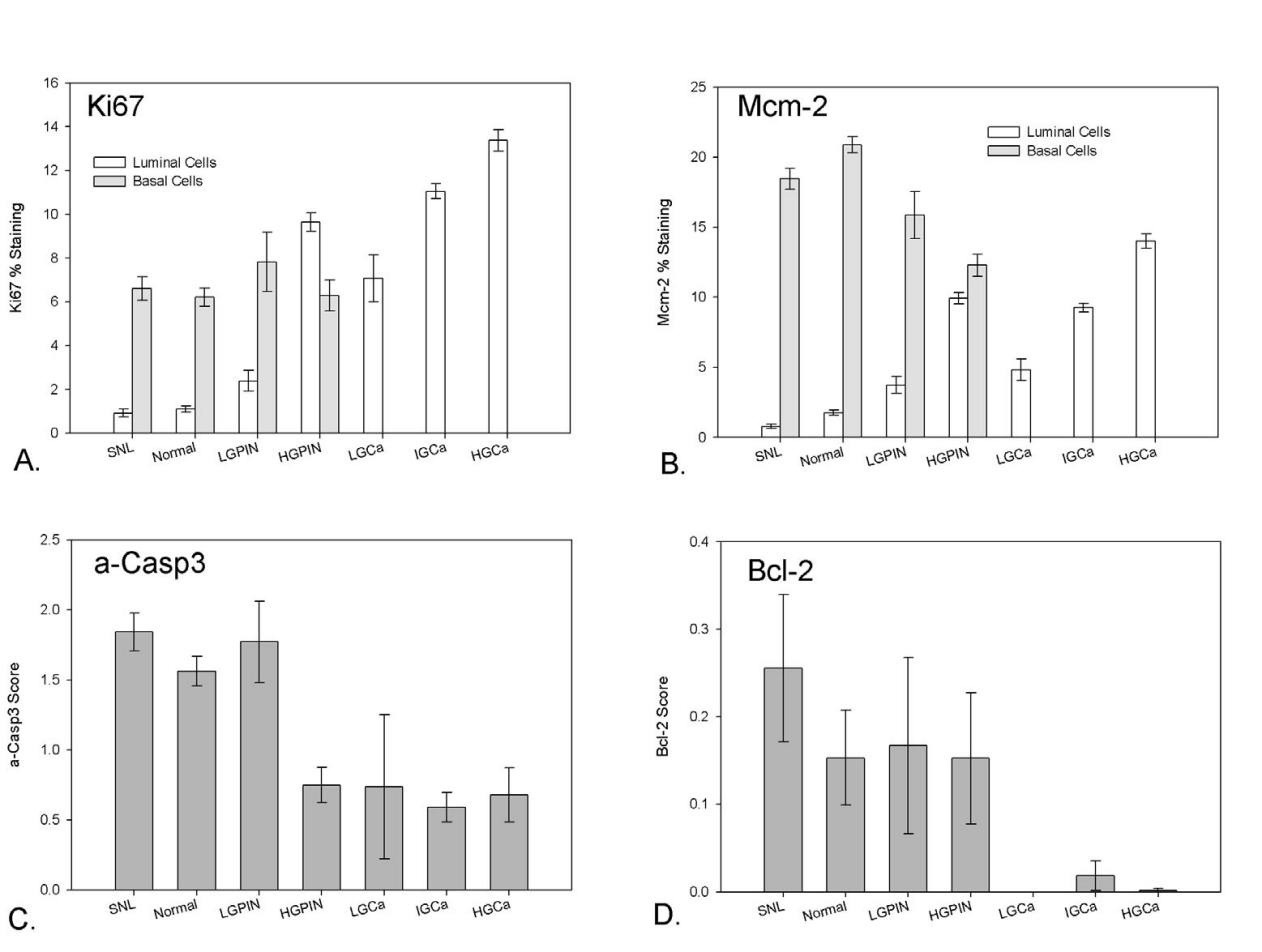
A and B show percent staining for Ki67 and Mcm-2 with 95% confidence intervals across progressive tissue compartments. There is a strong shift in proliferation from basal to luminal cell compartments in both markers. C and D show a-casp3 and Bcl-2 scores with 95% confidence intervals. Only the luminal compartment was scored for these 2 markers. For a-casp3 a sharp significant drop in expression was seen in HGPIN and cancer compartments. No differential trend was observed for Bcl-2. Supernormal glands showed significantly higher luminal Mcm-2 indices and higher a-casp-3 activity than normal glands suggestive of a field effect.

Supernormal and normal glands showed a preponderance of Ki67 and Mcm-2 positive nuclei in the basal sub-compartment, as noted previously and as shown in Figure 1.[9,10] Mcm-2 basal cell indices were significantly higher than Ki67 basal cell indices. The luminal, differentiated cells of the normal and supernormal glands had lower indices for both proliferation markers. Both Ki67 and Mcm-2 showed an upward trend from normal tissue through HGPIN to cancer (Fig. 2: panels A, B). Within the cancer compartment, both Ki67 and Mcm-2 increased significantly from low- to intermediate- to high-grade cancer. Overall, Mcm-2 indices were higher than Ki67 indices. In addition to the upward trend with progression in the non-cancer areas, a shift in proliferation from the basal cell compartment to the luminal cell compartment was noted. There was a significant correlation between total Ki67 and Mcm-2 indices measured in the same normal areas (Spearman R = 0.53, p = 0.001). The Ki67: Mcm-2 correlation for the luminal and basal cell sub-compartments were also significant (Spearman R = 0.69, p < 0.0001 and Spearman R = 0.42, p = 0.011, respectively).

#### Activated caspase-3 and Bcl-2

As expected, a-casp3 demonstrated uniform and homogenous cytoplasmic staining (Fig. 1) in the luminal cells of the normal glands.[11] A significant decrease in a-casp3 expression was noted in HGPIN and cancer glands when compared to normal glands (Fig. 2: panel C). LGPIN did not show any significant difference in expression from normal or supernormal glands. Within the cancer compartment there was no specific pattern associated with grade. Virtually all the non-cancerous glands showed consistent Bcl-2 staining in the basal cell sub-compartment. Luminal cell staining for Bcl-2 was sparse with extremely low numerical values on a 0–3 scale across all compartments.

### Evidence for field effects in prostatic carcinogenesis

#### Field effects by "near-far" comparison

Proliferative activity as measured by Mcm-2 expression was significantly higher in normal glands near cancer than ones that were more distant. This was reflected by significantly higher luminal and total Mcm-2 indices as well as higher luminal to basal ratios (see Table 2). However, Ki67 did not show a significant difference between "near" and "far" glands for the normal compartment. With regards to apoptosis-related markers, normal "near" glands demonstrated a higher a-casp3 activity than "far" normal glands. No "near-far" difference for Bcl-2 expression was noted. "Near" HGPIN glands showed a high-risk profile in terms of both proliferation and apoptotic markers. Specifically, HGPIN foci near cancer had higher Ki67 and Mcm-2 luminal and total indices, higher luminal to basal ratios, significantly lower a-casp3, and higher Bcl-2 expression when compared to HGPIN glands that were distant (see Table 2).

#### Field effects: normal versus supernormal gland comparison

**Figure 3:**
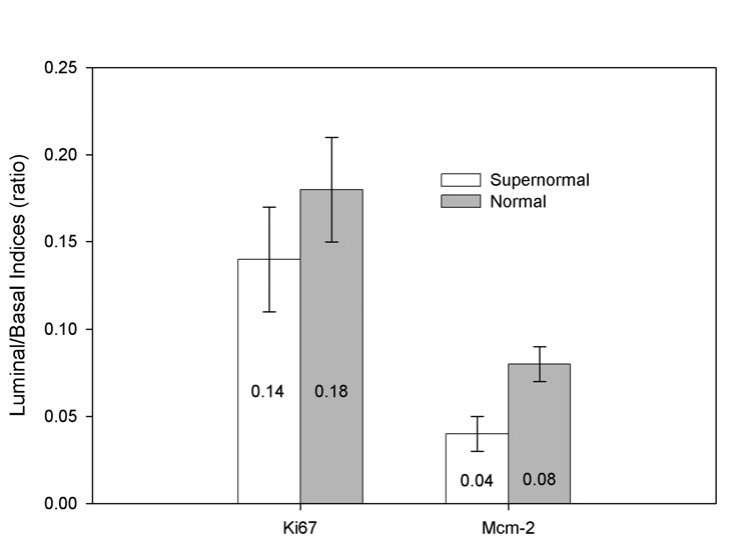
Figure 3 shows the luminal to basal cell ratio for the supernormal (normal glands from prostates without cancer) and normal (normal glands from prostates with cancer) compartments. Both markers showed a significantly higher luminal: basal ratio in the normal compartment.

Normal glands from prostates without foci of cancer had significantly lower Mcm-2 indices when compared to normal glands from prostates containing foci of cancer (see Fig. 2B). Also, the luminal to basal cell ratios for Ki67 and Mcm-2 were significantly lower in the supernormal glands (Fig. 3). Mean a-casp3 expression in the normal glands (1.56, 95% CI: 1.45, 1.67) was significantly lower than supernormal glands (1.84, 95% CI: 1.71, 1.98), consistent with a field effect involving lower apoptosis in high-risk tissue. Staining for the anti-apoptotic marker Bcl-2 in the luminal compartment was actually higher in supernormal compared to normal tissue (Fig. 2D). Foci of HGPIN from cystoprostatectomy specimens did not show any difference in the biomarker expression profile compared to HGPIN foci obtained from radical prostatectomy samples (data not shown).

## Discussion

In this study, both proliferation markers showed a progressive increase in positivity from histologically normal prostatic glands to HGPIN to cancer. In addition, a strong shift from basal to luminal epithelial layer proliferation was apparent early in the progression to neoplasia. In terms of field effects, we found that normal glands and HGPIN glands located near foci of cancer had increased proliferative activity, as indicated by higher Mcm-2 indices, when compared to equivalent glands that were distant. Further supporting the concept of field effects in the prostate, we found that normal glands from prostates free of cancer (i.e., supernormal) had lower proliferation activity when compared to normal glands from prostates that harbored cancerous foci. The luminal to basal cell ratio for Ki67 and Mcm-2 was significantly lower in the supernormal glands compared to the normal glands, consistent with the hypothesis that alteration in the luminal to basal cell proliferation ratio is an important early event in prostatic carcinogenesis. In fact, luminal to basal ratio, using either Ki67 or Mcm-2, was the strongest discriminator between supernormal and normal glands. Our results for the apoptosis markers were mixed. Expression of activated caspase 3 decreased with progression and was higher in supernormal tissue; however, in prostates containing cancer, a-casp3 activity appeared to be higher in normal areas near tumor foci. The apoptosis inhibitor Bcl-2, did not show a differential trend across pre-cancer compartments; nor did it show expression patterns suggestive of field effects in normal tissue. However, HGPIN areas near cancer foci had expression patterns indicating more inhibited apoptosis than HGPIN areas that were distant.

HGPIN lies intermediate in the morphologic continuum between benign and carcinomatous glands, and many biomarkers are either upregulated or downregulated in this neoplastic progression pathway.[12] Our observation that Ki67 expression in HGPIN lesions was intermediate when compared to normal and cancer, is consistent with previous studies.[9,13-15] Higher basal Ki67 indices for the normal compartment in our data could be due to more detailed scoring of basal cells at higher magnification and to the use of amplification methods to enhance the sensitivity for detecting weak immunohistochemical signals.

In this study, Mcm-2, a protein belonging to the Minichromosome Maintenance Protein (Mcm) family, was shown to be advantageous as a proliferation marker in several respects. Mcm proteins, which are required for DNA replication in all eukaryotic cells,[16] form a pre-replicative complex by binding to specific DNA sites. These complexes facilitate DNA replication and restrict replication to once-per-cell cycle.[17] Mcm-2 and Ki67 were strongly correlated when comparing the same tissue areas from the normal compartment. As noted in studies largely performed on non-prostate tissue, the proportion of cells positive for Mcm-2 was much higher.[9,17-19] Meng et al. found Mcm-2 expression in non-malignant prostate glands to be lower than we did (< 2%); however, this difference could be due to a number of variables in tissue preparation and analysis, including use of a different antibody clone.[10] Transition from basal to luminal proliferation with progression was also more evident with Mcm-2 than with Ki67. In contrast to Ki67, which has an undefined role in the cell cycle,[20] Mcm-2 plays a central role in chromatin replication [21], and has a longer half-life in replicating cells. Therefore, Mcm-2 could be more suitable for evaluation of normal tissue with low rates of proliferation, or in biopsy or cytology samples where tissue material is relatively sparse. A low proliferative index, such as one observes for Ki67 in normal prostate, requires counting a large number of cells in order to achieve acceptable statistical precision.

Another proliferation marker known to be differentially expressed in normal, HGPIN and cancer,[22,23] is proliferating nuclear cell antigen (PCNA). Antibodies against PCNA work only on paraffin-embedded tissues, [17] and detect cells undergoing DNA repair,[24] in addition to proliferating cells. Mcm proteins, on the other hand, are more specific markers for proliferation and are able to detect proliferating cells in both frozen and paraffin tissues.

Apoptotic stimuli activate initiator proteases such as caspase 8 and 9; these in turn activate executioner caspases, including caspase 3, which is the final link in the apoptotic signal cascade.[25,26] Although our data provide some support for activated caspase 3 as a marker of apoptotic activity in pre-cancerous tissue, the results were not entirely consistent because expression was actually higher in normal areas adjacent to cancer foci. Some studies have observed that increased apoptotic activity is linked with increased proliferation in cancer.[27,28] This phenomenon, which could be due to aberrant cell replication followed by programmed cell death in more rapidly growing tissue, could explain our results, which suggest that decreased apoptotic activity is an indicator of high-risk normal tissue only in the presence of low proliferative activity. This hypothesis will require further study. Although activated or cleaved caspase 3 is an attractive candidate marker for apoptotic activity, data on its expression in the prostate are still relatively sparse. Winter et al., in their study on caspase 1 and caspase 3 in the prostate, found that caspase 3 expression was reduced in moderately- and poorly-differentiated prostatic tumors compared to well-differentiated carcinomas and normal prostate.[11] Sohn et al. found that 42.5% of their cases of benign prostatic hyperplasia met the criteria to be scored as positive for caspase 3 versus only 28.6% of their grade III (Gleason score 8–10) cancers.[29] Similarly, O' Neill et al. found a significant decrease in expression of caspase 3 in high-grade cancer compared to BPH.[30] To our knowledge, the present study is the first to report decreased expression of activated caspase 3 staining in HGPIN lesions as compared to normal glands. However, decreased caspase 3 staining has been described in cervical intraepithelial neoplasia when compared to normal cervical epithelium.[31]

The TUNEL assay – a widely used assay for estimation of apoptosis – shows a progressive decrease in positive cells from normal to HGPIN to cancer.[8] However, there are specificity concerns with this method – areas of necrosis or autolysis and non-apoptotic nuclei showing signs of active gene transcription can produce false positive labeling. Variation in fixation, processing techniques, and proteinase digestion can also be a source of significant error.[32-34] Direct comparison of TUNEL to activated caspase 3 in prostate cancer xenografts has shown better agreement for caspase 3 immunostaining with time-consuming morphological identification of apoptotic cells.

Our findings suggest that, while the apoptosis inhibitor Bcl-2 might be a useful marker of HGPIN that is spatially associated with cancer, it does not however, appear to be a useful marker of high-risk normal tissue. Previous studies have suggested that Bcl-2 could be a promising marker for early prostate carcinogenesis. Using a dichotomous scoring criteria, Baltaci et al. reported Bcl-2 expression in 12 of 15 HGPIN lesions and 12 of 18 LGPIN lesions, with staining present in both basal and luminal layers, as opposed to basal staining only in BPH samples.[35] Johnson et al. found Bcl-2 overexpression in 34.9% cases of HGPIN.[13] In our study, there were essentially no differences in Bcl-2 across the pre-cancer tissue compartments.[13,36,37] The basal cell compartment and the positive control sample (tonsil) displayed consistently strong expression of Bcl-2, indicating that assay conditions were optimized. This basal cell expression is consistent with a prolonged lifespan and possible stem cell function of the basal cells.[36,38] Due to the lack of variation in Bcl-2 expression in the basal layer, we scored only the luminal layer. Since luminal expression in benign glands was minimal or absent, there was little dynamic range in the Bcl-2 score and, therefore, low reliability can be expected unless much larger amounts of tissue are evaluated.

Field cancerization, as described by Slaughter et al. and expanded upon by others, is the development of genetic or epigenetic damage in normal-appearing mucosa consequent to the exposure of an entire epithelial field to carcinogens.[3] Field effects have been implicated in the recurrence of tumors and also in the development of second primary tumors. Field cancerization in the prostate could be particularly useful in characterizing molecular signatures of apparently normal-looking mucosa adjoining a focus of cancer. Analysis of key cellular events such as loss of heterozygosity, allelic imbalance and methylation abnormalities have been used to address the issue of field effects at other anatomical sites.[39-41] Surprisingly, there are very few studies on field effects in the prostate. In the prostate, immunohistochemical evidence supporting field effects has been shown for diverse markers including Alpha Methylacyl-CoA Racemase (AMACR) – an enzyme involved in branched chain fatty acid metabolism,[42,43] EPCA – a nuclear matrix protein[44], Akt-1 – a cell survival molecule[45], and pS2 – an estrogen inducible protein.[46] Montironi et al. found lower expression of glutathione S-transferase-π and higher expression of telomerase activity in normal tissue adjoining neoplastic or pre-neoplastic lesions.[47] Yu et al. found 1022 genes that were differentially expressed in prostates adjacent to cancer when compared with organ donor prostates. Moreover, 70% of the genes were similarly altered in tumor samples and prostates adjoining tumor, suggesting a general similarity of expression patterns between the two.[43] Nuclear morphometry studies in prostate have demonstrated chromatin distribution abnormalities and subtle changes in apparently normal-looking nuclei.[47-49] Such alterations have been noted for a distance of 10 mm from the margin of a either a PIN or an adenocarcinoma focus.[49]. Montironi.et al. reported that PIN lesions from cystoprostatectomy specimens had significantly lower mean nuclear and nucleolar area when compared to similar lesions from radical prostatectomy specimens. This study also found, in agreement with our results, that Ki67 indices in PIN lesions from cystoprostatectomy and radical prostatectomy specimens were not meaningfully different.[50].

Proliferation markers have been used to study field cancerization at other sites. In their study on hamster cheek pouch carcinogenesis model, Raimondi et al. found that ploidy values and 5-bromo-2-deoxiuridine (BrdU) were higher in carcinogen-exposed epithelia with no unusual microscopic features when compared to control epithelia.[51] Similarly, Barsky et al. found that Ki67 indices in normal bronchial mucosa of smokers were higher than that of non-smokers.[52] We are not aware of previous reports on the use of caspase 3 or any other apoptotic marker for the evaluation of field effects.

In summary, we demonstrate that biomarkers identifying key cellular events like proliferation and apoptosis are altered not only in preneoplastic lesions but also in apparently normal-looking epithelium. Data obtained from normal tissues adjoining a prostate cancer focus need to be interpreted in the light of the fact that these tissues are subject to field effects. We conclude that Mcm-2 could be superior to Ki67 for detection of subtle field effects, and that activated caspase-3 shows some promise as an indicator of high-risk normal tissue. The implication, however, that normal areas immediately adjacent to cancer might have increases in both proliferation and apoptosis compared to distant areas suggests that the balance between these two processes rather than their absolute levels could be important or, to state it differently, the significance of the level of apoptosis in an area depends on the level of proliferation, and vice-versa. Future plans call for implementation of a multivariate model comprised of various biomarkers and clinical variables to predict outcomes following negative biopsies in a larger study set.

## Abbreviations

A-casp3, activated caspase-3; Bcl-2, B-Cell Leukemia/Lymphoma-2; BPH, benign prostatic hyperplasia; CP, cystoprostatectomy; DAB, Diaminobenzidine; H&E hematoxylin and eosin; HGCA, high-grade cancer; HGPIN, high grade prostatic intraepithelial neoplasia; IGCA, intermediate-grade cancer; LGCA, low-grade cancer; LGPIN, low grade prostatic intraepithelial neoplasia; Mcm-2, Minichromosome Maintenance Protein-2; RP, radical prostatectomy

## Competing interests

The author(s) declare that they have no competing interests.

## Authors' contributions

VA read out the H&E slides, scored the immunostained slides and drafted the manuscript. RJD carried out the data analysis. XYJ and MRP were involved in expert classification of the various histopathological lesions. PHG conceived the study, participated in its design and coordination and helped to draft the manuscript. All authors read and approved the final manuscript.

## Pre-publication history

The pre-publication history for this paper can be accessed here:



## Figures and Tables

**Table 1:** Number of patients, cells, and 40 × microscopic fields evaluated for expression of Ki67, Mcm-2, Bcl-2, and activated caspase 3 (a-casp3)

Tissue Compartment	Number of Patients				Number of Cells		Number of Fields	
	
	Ki67	Mcm-2	a-Casp3	Bcl-2	Ki67	Mcm-2	a-Casp3	Bcl-2
HGCa	7	7	7	7	18,279	17,366	71	85
IGCa	12	12	12	11	33,302	32,735	165	195
LGCa	3	3	3	3	2,219	3,130	10	15
HGPIN	12	12	13	13	23,054	27,895	188	191
LGPIN	8	8	10	10	5,300	5,561	52	44
Normal	12	12	12	12	36,141	38,542	218	224
Supernormal	6	6	6	6	20,901	21,224	145	157

**Table 2:** Ki67, Mcm-2, a-Casp3, and Bcl-2 scores in the near (<1 mm from tumor) and far (>5 mm from tumor) areas of the normal and HGPIN compartments. Bold numerals indicate *P *value < 0.05 for difference between near and far glands.

	Normal		HGPIN	
	
	Near mean (95% CI)	Far mean (95% CI)	Near mean (95% CI)	Far mean (95% CI)
Ki67 (% positive)				
Luminal	1.25 (0.98,1.52)	1.18 (0.88,1.48)	**10.77 **(10.05,11.50)	6.43 (5.48,7.38)
Basal	6.47 (5.72,7.23)	6.29 (5.41,7.16)	6.12 (4.82, 7.41)	6.85 (5.02,8.68)
Total	3.27 (2.94,3.61)	3.09 (2.71,3.47)	**10.04 **(9.40,10.69)	6.52 (5.68,7.37)
Lum:Basal Ratio	0.19 (0.15,0.25)	0.19 (0.14,0.25)	**1.76 **(1.41, 2.20)	0.94 (0.69,1.27)
Mcm-2 (%positive)				
Luminal	**2.17 **(1.78,2.56)	1.61 (1.26,1.95)	**14.75 **(13.84,15.67)	7.66 (6.74,8.59)
Basal	22.45 (21.33,23.56)	22.80 (21.46,24.15)	**12.91 **(11.10,14.72)	16.93 (14.80,19.07)
Total	**12.24 **(11.62,12.86)	10.62 (9.98,11.27)	**14.41 **(13.59,15.23)	10.20 (9.30,11.10)
Lum:Basal Ratio	**0.10 **(0.08,0.12)	0.07 (0.06,0.09)	**1.14 **(0.98,1.33)	0.45 (0.38,0.54)
a-casp3^a^	**1.72 **(1.57,1.52)	1.52 (1.38,1.66)	**0.60 **(0.46,0.74)	0.88 (0.67,1.08)
Bcl-2^a^	0.11 (0.04,0.19)	0.13 (0.05,0.22)	**0.19 **(0.07,0.31)	0.05 (0.00,0.11)

^a ^For cytoplasmic markers, the numbers in the table represent mean staining intensity in luminal cells only, scored as 0–3 per 40 × field.
